# Potential for distinguishing the parkinsonian subtype of multiple system atrophy from Parkinson’s disease: a three-dimensional gait analysis study

**DOI:** 10.3389/fnagi.2026.1797960

**Published:** 2026-05-07

**Authors:** Reyisha Taximaimaiti, Sheng Cai, Hongyan Li

**Affiliations:** 1 Department of Neurology, People's Hospital of Xinjiang Uygur Autonomous Region, Urumqi, Xinjiang, China; 2 College of Life Sciences and Technology, Xinjiang University, Urumqi, Xinjiang, China; 3 Zhejiang Province Key Laboratory of Anti-Cancer Drug Research, College of Pharmaceutical Sciences, Zhejiang University, Hangzhou, Zhejiang, China

**Keywords:** cognitive load test, endogenous beat test, gait analysis, multiple system atrophy, Parkinson’s disease, TUG test, wearable sensors

## Abstract

**Objective:**

Distinguishing the parkinsonian subtype of multiple system atrophy (MSA-P) from Parkinson’s disease (PD) at an early stage was a challenge, and gait analysis based on wearable sensors offers a reliable method to quantify gait parameters. In this study, we used a three-dimensional gait motion capture system based on wearable sensors to identify potential gait-based biomarkers that might assist in distinguishing MSA-P from PD.

**Methods:**

Participants were enrolled in this observational study from the Department of Neurology at the People’s Hospital of Xinjiang Uygur Autonomous Region from June 2024 to October 2025 and were required to complete the timed up and go (TUG) test, Cognitive Load Test, and Endogenous Beat Test according to our instructions. Multivariate analysis of covariance (MANCOVA) was used to conduct overall comparisons of gait characteristics among groups, and predictive ability was tested through 5-fold cross-validated receiver operating characteristic curves and sensitivity analysis.

**Results:**

A total of 74 PD patients, 33 MSA-P patients, and 79 healthy controls (HCs) were enrolled in this study. When PD patients and MSA-P patients exhibited similar clinical performance and drug dosage, MSA-P patients had a 4.9-year younger age of symptom onset and a duration of symptoms that was 19.4 months shorter. Both PD patients and MSA-P patients exhibited reduced stride length, reduced velocity, reduced bilateral step length, anterior pelvic tilt, and broadly reduced range of motion (ROM) of the pelvis and the lower limb joints compared to HCs. The negative impact of the Cognitive Load Test on gait seemed to be far greater than that of the Endogenous Beat Test, and PD patients might be more sensitive to the Endogenous Beat Test than MSA-P patients. The ROM of hip flexion–extension in the Endogenous Beat Test demonstrated the most favorable discriminative and potential predictive capacity for distinguishing MSA-P patients from PD patients, and the results were stable across different subgroups of patients according to sensitivity analysis.

**Discussion:**

The ROM of hip flexion–extension in the Endogenous Beat Test might have a potential capacity to distinguish MSA-P patients from PD patients. Three-dimensional gait analysis based on wearable sensors might hold considerable application potential for the clinical differential diagnosis of neurodegenerative diseases.

## Introduction

Parkinson’s disease (PD) and multiple system atrophy (MSA) are among the most common *α*-synucleinopathies, which are often characterized by refractory gait disturbance and lifelong disability (Shanshan et al., 2024; Xiao et al., 2024). PD can be classified into Park Sleep, cholinergic, and noradrenergic subtypes, and cues such as horizontal stripes have been used to enhance walking performance for cholinergic subtypes (Xiao et al., 2024). MSA can be classified into the parkinsonian subtype of MSA (MSA-P) and the cerebellar subtype of MSA (MSA-C) according to predominant initial symptoms and is characterized by aggressive progression with an average survival of 8–9 years after symptom onset, which is generally shorter than that of PD (Florian et al., 2024). Currently, distinguishing MSA-P from PD at early stages remains challenging, largely due to their overlapping clinical and pathological presentations (Mohammad et al., 2020). Although various approaches have been explored to identify potential markers for differentiating MSA-P from PD and have yielded some encouraging findings, the results have often been inconsistent across different studies, and suboptimal sensitivity may limit their broader clinical application (Florian et al., 2024; Ma et al., 2024; Annika et al., 2024). Accordingly, identifying biomarkers that can conveniently distinguish between these two diseases remains an important unmet need.

Wearable sensor technology, as a notable example of digital health technologies, has successfully translated into clinical practice and has received sustained attention due to its many advantages, such as non-invasiveness, simplicity of operation, and cost-effectiveness (Farwa et al., 2025). Gait analysis based on wearable sensors has been proven to contribute to the management of neurodegenerative disease, supporting clinical diagnosis, disease progression tracking, disease rehabilitation, stepped care, and future outcome prediction (Farwa et al., 2025; Poplawska-Domaszewicz et al., 2025). With wearable sensors placed on limbs or the trunk, a real-time three-dimensional gait motion capture system can provide spatiotemporal and kinematic data during walking, offering a reliable method to identify abnormal gait patterns and objectively quantify gait parameters in neurodegenerative diseases (Martin et al., 2022).

In previous studies, PD patients have been observed to have some typical abnormal gait patterns, such as reduced walking speed and step length, increased cadence, reduced swing time, impaired rhythmicity, and increased axial rigidity (Grazia et al., 2021). MSA patients display high gait variability, increased axial rigidity, and increased postural instability (Victoria et al., 2021). Based on these results, targeted rehabilitation programs (e.g., virtual reality [VR] gait training, treadmill training, and robot-assisted gait training) have shown potential benefits for improving gait stability, balance, and gait symmetry (Yong et al., 2022). However, the detailed comparison of gait characteristics between PD patients and MSA-P patients remains inadequate, and the identification of valid gait-based biomarkers to support early clinical diagnosis still remains a critical clinical challenge.

In this study, we used a three-dimensional gait motion capture system based on wearable sensors to evaluate the gait characteristics of PD patients, MSA-P patients, and healthy controls (HCs) across different gait tests. We aimed to identify potential gait-based biomarkers that might assist in differentiating MSA-P from PD and provide a basis for the development of digital mobility assessment tools that could potentially guide differential diagnosis and personalized rehabilitation interventions in the future.

## Methods

### Participants

**Figure 1:**
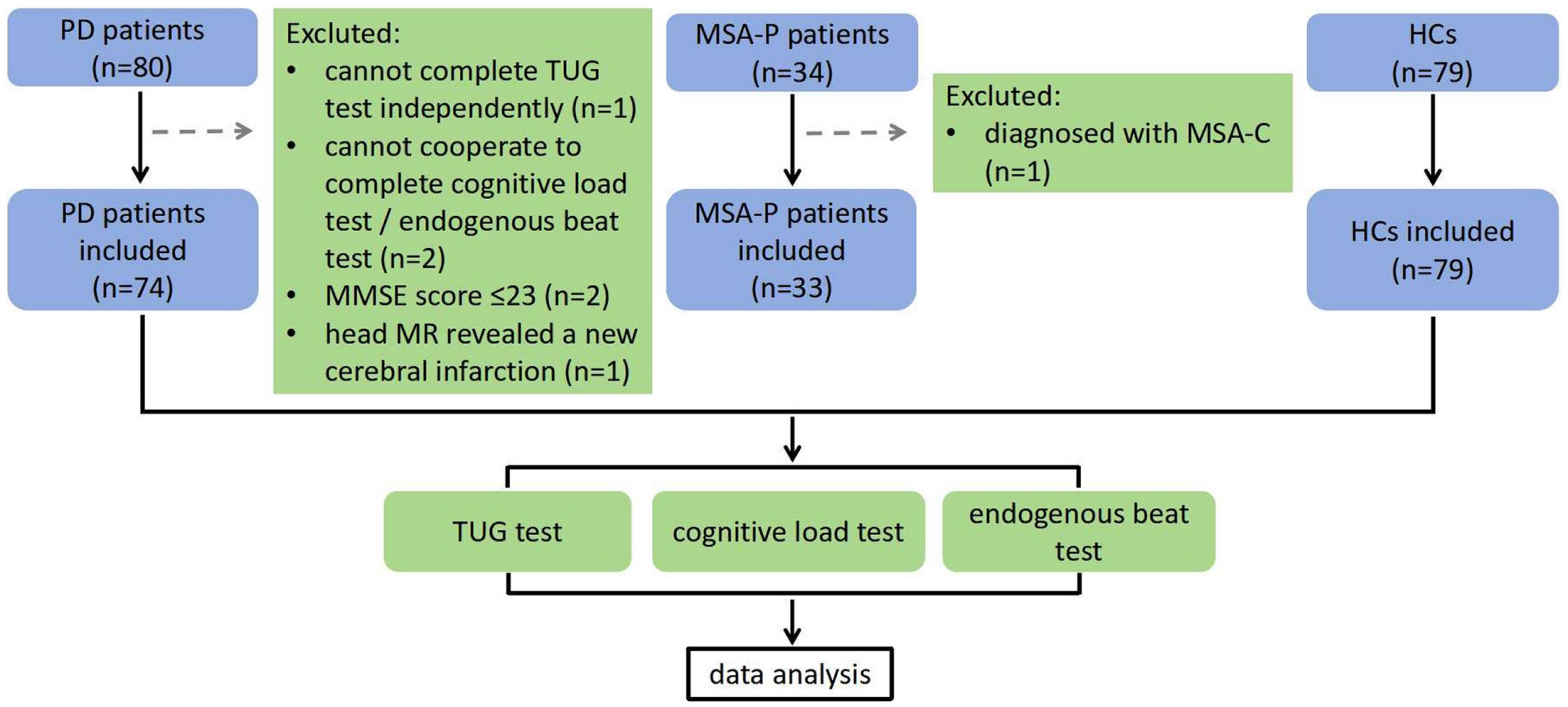
Flowchart of inclusion and exclusion. PD: Parkinson’s disease, MSA-P: the Parkinsonian subtype of multiple system atrophy, MSA-C: the cerebellar subtype of multiple system atrophy, HC: health control, TUG: timed up and go, MMSE: Mini-Mental State Examination.

From June 2024 to October 2025, 74 PD patients, 33 MSA-P patients, and 79 HCs were recruited from the Department of Neurology at the People’s Hospital of Xinjiang Uygur Autonomous Region (Figure 1). All gait assessments were conducted in the testing area on the 10th floor of building 4 of the aforementioned hospital. All participants underwent a comprehensive neurological and neuropsychological examination before the tests. The diagnosis of idiopathic PD and MSA-P was confirmed by two experienced neurologists specializing in movement disorders, according to validated clinical diagnostic criteria for PD (Hughes et al., 1992) and MSA (Wenning et al., 2022). MSA-P patients were classified according to the predominant symptom of Parkinsonism at the time of initial diagnosis and at the time when this study was carried out.

The inclusion criteria for PD patients and MSA-P patients included (1) 40–85 years of age; (2) clinical observations showed typical Parkinsonism syndrome; (3) both lower limbs were intact, with no obvious deformity; (4) no knee surgery within the past 6 months; (5) no history of other neurological diseases, hereditary disorders, or serious psychiatric diseases; (6) ability to complete the timed up and go (TUG) test independently; (7) Mini-Mental State Examination (MMSE) score >23; and (8) capability of cooperating in completing the gait tests required for this study. The inclusion criteria for HCs included: (1) no history of movement disorders, (2) no family history of movement disorders, and (3) all other criteria were identical to those for PD and MSA-P patients.

### Data source

Demographic information was collected from all participants, and clinical information, including Unified Parkinson’s Disease Rating Scale-part III (UPDRS-III) score, Hoehn–Yahr score, and levodopa equivalent daily dose (LEDD), was collected from PD patients and MSA-P patients according to their medical history records and patient reconfirmation.

**Figure 2:**
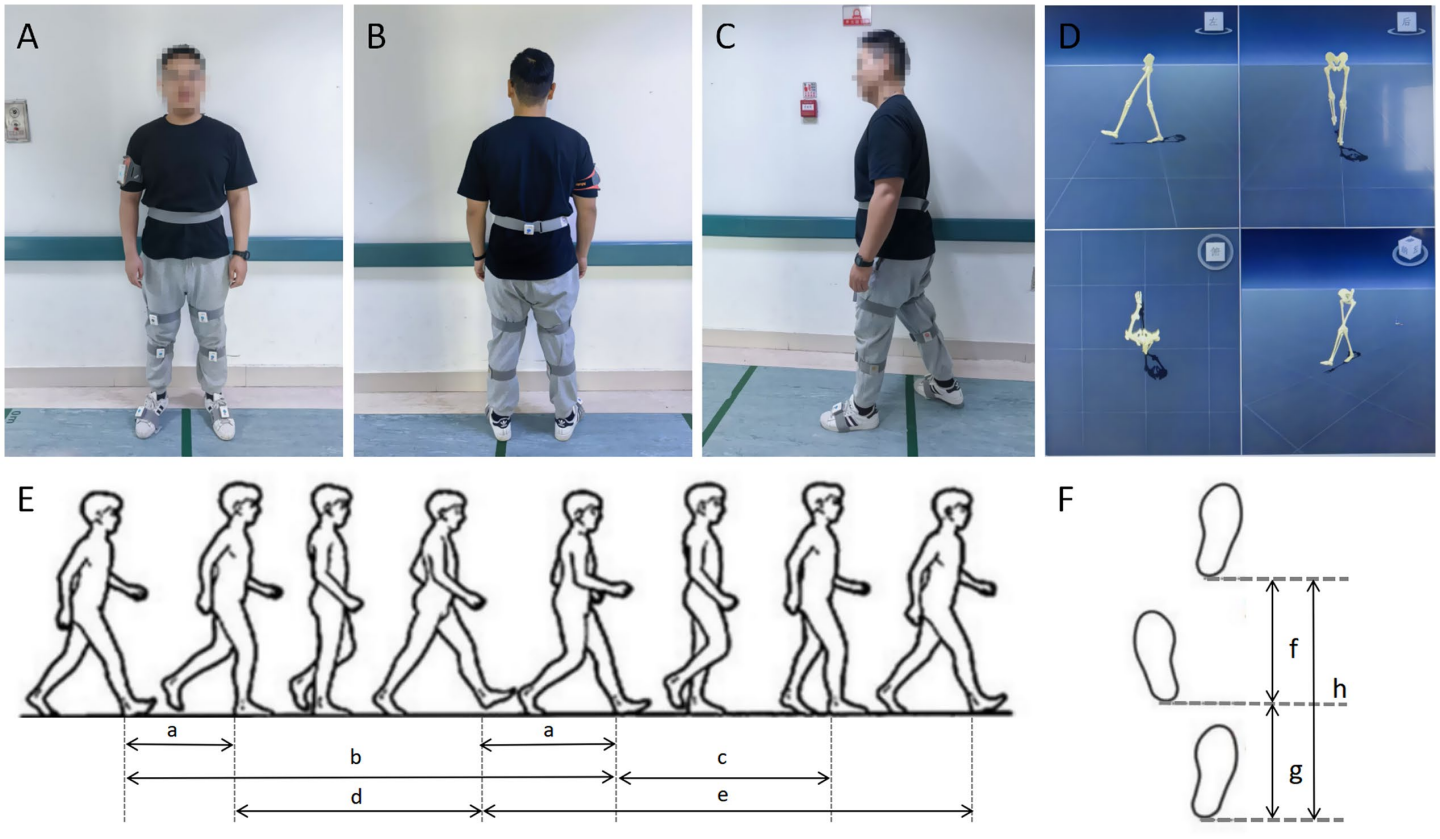
Diagram of the gait characteristics acquisition process. **(A–C)** The sensor placement position of the GaitWatch three-dimensional gait motion capture system. The sensors were placed in the following positions: the midpoint of the posterior waist at the level of the navel, the mid-anterior sides of the left and right thighs (10 cm above the knees), the mid-anterior sides of the left and right calves (10 cm below the knees), and the mid-dorsal tops of the left and right feet. The main controller was placed on the right upper arm. **(D)** The test interface of the GaitWatch three-dimensional gait motion capture system. Images of the participant’s pelvis and lower limbs while walking were displayed on the screen: the left sagittal plane, the posterior coronal plane, the top-down horizontal plane, and the panoramic view. **(E)** Diagram of gait. a: double stance phase, b: right stance phase, c: right swing phase, d: left swing phase, e: left stance phase. **(F)** Diagram of footsteps. f: right step length, g: left step length, h: stride length.

*GaitWatch,* a three-dimensional gait motion capture system based on wearable sensors, was used to capture gait characteristics. The sensors were placed at the midpoint of the posterior waist at the level of the navel, the mid-anterior sides of the left and right thighs (10 cm above the knees), the mid-anterior sides of the left and right calves (10 cm below the knees), and the mid-dorsal tops of the left and right feet, respectively. The main controller was placed on the right upper arm, as shown in Figure 2. Before the start of each gait test, the researcher checked the positions of all sensors to ensure that no sensor displacement occurred during the tests. When the subject was walking, this system could capture the movement of each joint in the lower limbs and display sagittal, coronal, and horizontal images on the screen. Before each test, participants were required to stand in a normal posture for 5 s, after which the system recognized this posture as the initial posture for this test and automatically calibrated the posture of the subsequent walking test. To avoid the influence of body position changes on joint movement data, only the walking process after getting up from the chair and before sitting down on the chair (including turning around) was included in the gait analysis of this study. Participants were allowed to practice beforehand to ensure they walked naturally.

PD patients and MSA-P patients were required to take anti-parkinsonism drugs regularly 1 week before the test, and all tests were conducted during their “On” phase to avoid fall events. After the researcher explained the test procedures and requirements, participants were first asked to complete the TUG test (with a total round-trip distance of 10 m) at a comfortable speed, which was set as the baseline. Next, participants were asked to complete the Cognitive Load Test: while completing the same TUG test, continuously subtracting 7 from 100 (“100−7=?, −7=?, −7=?, …”), and speaking the answers loudly. At least two calculations completed in one test were confirmed as valid, regardless of whether the results were correct or not. Next, participants were asked to complete the Endogenous Beat Test (Elinor et al., 2025a): while completing the same TUG test, producing an internal rhythmic cue (e.g., repeatedly saying “121, 121, …” aloud) and keeping the pace and rhythm in harmony. Each test was repeated three times, and the average value was recorded for analysis. No instruction was given to start walking with a specific leg to ensure that participants could randomly choose either leg to start with in each trial.

Gait analysis based on wearable sensors included spatiotemporal parameters (cadence, gait cycle, step length, velocity, stance phase, and swing phase) and kinematic parameters of the pelvis and joints in the lower limbs (hip, knee, and ankle) (Grazia et al., 2021). Cadence refers to the average number of steps per minute. Velocity refers to the average distance walked per second. Step length refers to the average distance from one foot touching the ground to the other foot touching the ground while walking. Stride length refers to the average distance from one foot touching the ground to the same foot touching the ground again while walking, and it is also the sum of the step lengths of the left and right sides. Bilateral step length deviation refers to the difference in step length between the left and right sides. Gait cycle refers to the average time to complete a full stride. The stance phase refers to the average time when the foot is in contact with the ground (approximately 60% of the gait cycle), while the swing phase refers to the average time when the foot is off the ground (approximately 40% of the gait cycle) (Kalkhoran and Khanmohammadi, 2025). The pelvis’s average range of motion (ROM) was recorded in the sagittal plane (anteversion–retroversion), horizontal plane (axial rotation), and vertical movement, respectively. The knee’s average ROM was recorded in the coronal plane (flexion–extension). The ankle’s average ROM was recorded in the coronal planes (dorsiflexion–plantarflexion) and sagittal planes (inversion–eversion). We then identified the affected/dominant (A/D) side and the non-affected/non-dominant (non-A/non-D) side for every PD and MSA-P patient according to their performance in tests and their past medical history to conduct subsequent statistical analysis. For HCs, the A/D side and the non-A/non-D side refer to the non-dominant hand side and the dominant hand side, respectively.

### Ethics approval statement

The protocol of this study was approved by the Ethics Committee of the People’s Hospital of Xinjiang Uygur Autonomous Region (KY2025022107). Written informed consent was obtained from all participants. All processes of this study were carried out in accordance with the Declaration of Helsinki.

### Sample size evaluation

G*Power 3.1.9.7 was used to evaluate the sample size for this study. The MANOVA: Global effects using the power analysis type *a priori*: Compute required sample size, given the effect size f^2^ at 0.2, the power at 0.80, the *α* error probability at 0.05, the number of groups was 3, and the response variable was 1 (have PD or MSA-P), obtained a preliminary total sample size of 54. As three pairwise comparisons were required among the three groups (PD patients vs. HCs, MSA-P patients vs. HCs, and PD patients vs. MSA-P patients), the *post-hoc* Bonferroni-adjusted α value was 0.05/3 ≈ 0.017, resulting in a total sample size of 69. With 20% covariate influence and 15% sample attrition, at least 96 participants (32 participants in each group) were required to satisfy the actual power of the sample. The sample size of this study met the requirements.

### Statistical analysis

*GaitWatch,* a three-dimensional gait motion capture system based on wearable sensors (Zhanghe Intelligent, China), was used for gait analysis. Data statistical analysis was performed using SPSS 26.0 (IBM, United States) and R 4.5.1 (Posit Software, United States). Statistical plots were generated using Origin 2024 (OriginLab, United States), GraphPad Prism 10.1.2 (GraphPad Software, United States), and R 4.5.1. Quantitative variables were described as mean ± standard deviation (SD), and categorical variables were described as percentages (%). The Shapiro–Wilk test and analysis of variance (ANOVA) were used to assess the normality of data distribution and homogeneity of variance for quantitative variables. The χ2 test was used for comparisons of categorical variables.

When comparing demographic and clinical features, a one-way ANOVA was used to calculate overall comparisons among the groups, followed by post-hoc Bonferroni comparisons between the groups. For clinical quantitative indicators only for PD patients and MSA-P patients, a two-tailed independent samples t-test was used. When analyzing gait variables, Levene’s test was used to evaluate data normality, and a one-way multivariate analysis of covariance (MANCOVA) (adjusted for group, sex, age, body mass index [BMI], Mini-Mental State Examination [MMSE], Montreal Cognitive Assessment [MoCA], Hamilton Depression Scale [HAMD], and Hamilton Anxiety Scale [HAMA]) was used to conduct overall comparisons among the groups, followed by *post-hoc* Bonferroni comparisons between the groups and main effect tests. Next, the results of the TUG test were set as the baseline; ANCOVA was used to conduct overall comparisons, followed by post-hoc Bonferroni comparisons between the groups. Next, to evaluate the symmetry of gait, independent sample t-tests were used to compare gait variables on the A/D side and the non-A/non-D side.

Logistic analysis (adjusted for sex, age, BMI, symptom duration, UPDRS-III score, LEDD, MMSE, MoCA, HAMD, and HAMA) was used to explore the relationship between gait variables and the possibility of having MSA-P. Partial Pearson’s correlation analysis (adjusted for sex, age, BMI, symptom duration, MMSE, MoCA, HAMD, and HAMA) was used to evaluate correlations between clinical indicators and gait variables. Receiver operating characteristic (ROC) curve analysis was conducted to test the predictive ability of gait variables to identify MSA-P from PD. For those with statistically significant differences, a 5-fold cross-validation (repeated 100 times) was used for internal validation, and the area under the curve (AUC) and corresponding 95% confidence intervals (CIs) were calculated. Then, the results were further evaluated using decision curve analysis (DCA) curves and calibration curves. Next, we classified PD patients and MSA-P patients according to their sex, BMI, UPDRS-III score, and LEDD to set different models and conducted sensitivity analysis to verify the identification ability of gait variables. The optimal cutoff values for BMI, UPDRS-III score, and LEDD used in the sensitivity analysis were determined using the ROC curve analysis of PD and MSA-P patients by maximizing the Youden index (J = Sensitivity+Specificity-1). A two-tailed *p*-value < 0.05 was defined as statistically significant.

## Results

### MSA-P patients have a younger age of symptom onset and shorter symptom duration

Table 1 presents the demographic and clinical features of participants. Three groups had similar sex and BMI, while MSA-P patients had a younger age and longer education duration. When PD patients and MSA-P patients had similar clinical performance and drug dosage (UPDRS-III score, Hoehn–Yahr score, and LEDD), MSA-P patients had a 4.9-year younger age of symptom onset and a duration of symptoms that was 19.4 months shorter. PD patients and MSA-P patients had worse cognitive performance, Activity of Daily Living (ADL) Scale, and emotional state compared to HCs, while the conditions between these two groups were very similar.

**Table 1:** Demographic and clinical features of PD patients, MSA-P patients, and HCs.

Variables	PD patients (*n* = 74)	MSA-P patients (*n* = 33)	HCs (*n* = 79)	Overall comparison P	Between-group comparison P		
					PD vs. HCs	MSA-P vs. HCs	PD vs. MSA-P
Sex (male/female)	36/38	21/12	38/41	0.28	-	-	-
Age (years)	64.4 ± 8.2	58.8 ± 8.9	63.4 ± 8.4	**<0.01**	1.00	**0.03**	**<0.01**
Years of education (years)	9.2 ± 4.1	11.6 ± 3.5	9.3 ± 4.2	**0.01**	1.00	**0.02**	**0.02**
BMI (kg/m^2^)	25.3 ± 4.3	25.2 ± 3.5	26.8 ± 3.6	**0.04**	0.07	0.17	1.00
Age of symptom onset (years)	62.2 ± 10.0	57.3 ± 8.4	-	-	-	-	**0.02**
Symptom duration (months)	36.3 ± 36.1	16.9 ± 14.6	-	-	-	-	**<0.001**
UPDRS-III score	20.1 ± 9.8	19.7 ± 9.2	-	-	-	-	0.83
Hoehn–Yahr (1/2/3/4)	23/23/25/3	10/14/8/1	-	-	-	-	0.66
LEDD (mg/day)	391.1 ± 221.3	387.5 ± 200.1	-	-	-	-	0.94
MMSE	26.6 ± 1.9	26.8 ± 2.3	28.4 ± 1.8	**<0.001**	**<0.001**	**<0.001**	1.00
MoCA	22.6 ± 3.8	22.0 ± 4.0	25.5 ± 3.8	**<0.001**	**<0.001**	**<0.001**	1.00
ADL	26.7 ± 10.5	27.8 ± 9.7	21.0 ± 1.9	**<0.001**	**<0.001**	**<0.001**	1.00
HAMD	26.4 ± 4.6	26.2 ± 5.3	23.1 ± 3.5	**<0.001**	**<0.001**	**<0.01**	1.00
HAMA	20.7 ± 4.4	20.0 ± 5.3	17.3 ± 3.3	**<0.001**	**<0.001**	**<0.01**	1.00

PD: Parkinson’s disease, MSA-P: the parkinsonian subtype of multiple system atrophy, HCs: healthy controls, BMI: body mass index, UPDRS-III: Unified Parkinson’s Disease Rating Scale-part III, LEDD: levodopa equivalent daily dose, MMSE: Mini-Mental State Examination, MoCA: Montreal Cognitive Assessment, ADL: Activity of Daily Living Scale, HAMD: Hamilton Depression Scale, HAMA: Hamilton Anxiety Scale. One-way ANOVA was used to calculate the overall comparison P, and post-hoc Bonferroni was used to calculate between-group comparison P. *p* values with statistical significance are shown in bold.

### The ROM of hip flexion–extension in the endogenous beat test shows the best recognition ability to distinguish MSA-P from PD

**Figure 3:**
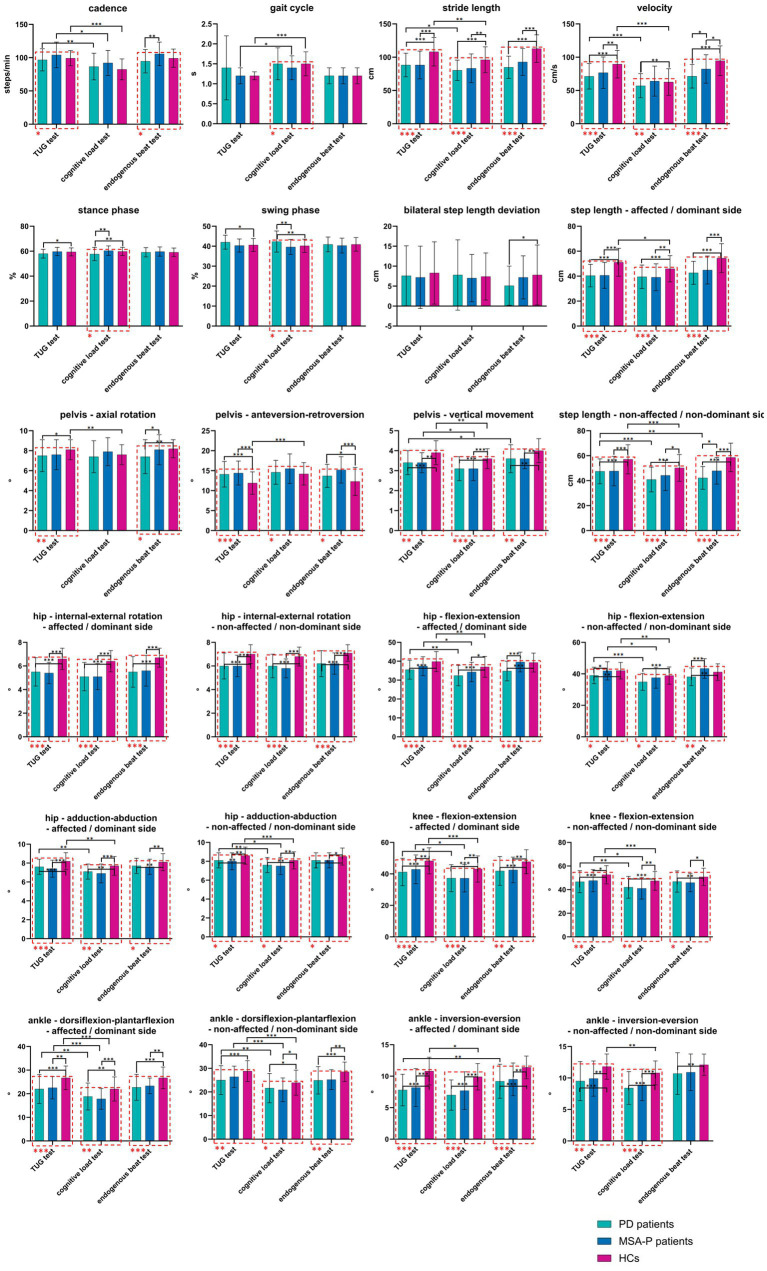
Gait characteristics of PD patients, MSA-P patients, and HCs in TUG test, cognitive load test, and endogenous beat test. PD: Parkinson’s disease, MSA-P: the Parkinsonian subtype of multiple system atrophy, HC: health control, TUG: timed up and go. MANCOVA (adjusted for group, sex, age, BMI, symptom duration, UPDRS-III score, LEDD, MMSE, MoCA, HAMD, and HAMA) was used to calculate overall comparisons *P*, and post-hoc Bonferroni was used to calculate between-group comparison *P*. The red dashed box indicated that the MANCOVA results suggested a statistically significant difference in the overall comparison among three groups. * indicated that there was a statistically significant difference in the between-group comparison. For details, please refer to Supplementary Tables 1, 3, 5, 7. *: *p* < 0.05; **: *p* < 0.01; ***: *p* < 0.001.

Both PD patients and MSA-P patients exhibited shorter stride length and bilateral step length, as well as smaller ROM in pelvic vertical movement, bilateral hip internal–external rotation, bilateral hip adduction–abduction, bilateral knee flexion–extension, ankle dorsiflexion–plantarflexion of the A/D side, and ankle inversion–eversion of the A/D side compared to HCs in all three tests. PD patients exhibited smaller ROM in hip flexion–extension on the non-A/non-D side than HCs in all three tests, while MSA-P patients did not exhibit smaller ROM. When comparing PD patients with MSA-P patients, PD patients exhibited smaller ROM in hip flexion–extension on the non-A/non-D side in the TUG test, shorter stance phase and longer swing phase in the Cognitive Load Test, and slower cadence and velocity, shorter step length on the non-A/non-D side, smaller ROM in axial rotation, and bilateral hip flexion–extension in the Endogenous Beat Test (Figure 3; Supplementary Tables 1, 3, 5). The main effect tests showed that group effects (PD/MSA-P/HCs) were the main reasons for these differences (Supplementary Tables 2, 4, 6). When setting the TUG test as the baseline, the Cognitive Load Test had a greater negative impact on gait than the Endogenous Beat Test (Supplementary Table 7). When evaluating gait symmetry (Supplementary Table 8), nearly all variables of the ROM of the lower limb joints and step length exhibited significant differences between the A/D side and the non-A/non-D side. The corresponding *F*-value suggested a trend that both the Cognitive Load Test and the Endogenous Beat Test tended to reduce bilateral asymmetry.

### The ROM of hip flexion–extension in the endogenous beat test shows the best predictive ability to distinguish MSA-P from PD

The results of logistic univariate and multivariate analyses (Supplementary Table 9) suggested that stance phase and swing phase in the TUG test, stance phase and swing phase in the Cognitive Load Test, and cadence, bilateral step length deviation, and the ROM of hip flexion–extension of the A/D side in the Endogenous Beat Test might be potentially useful for distinguishing MSA-P patients from PD patients. Partial Pearson’s correlation analysis in PD patients and MSA-P patients (Supplementary Figure 1) indicated that UPDRS-III was negatively related to velocity and step length of the A/D side in all three tests for PD patients, whereas no common gait variables demonstrating statistical significance were observed in Hoehn–Yahr scores and LEDD; and UPDRS-III, Hoehn–Yahr score, and LEDD were all negatively related to the ROM of hip internal-external rotation of the A/D side in all three tests in MSA-P patients.

**Figure 4:**
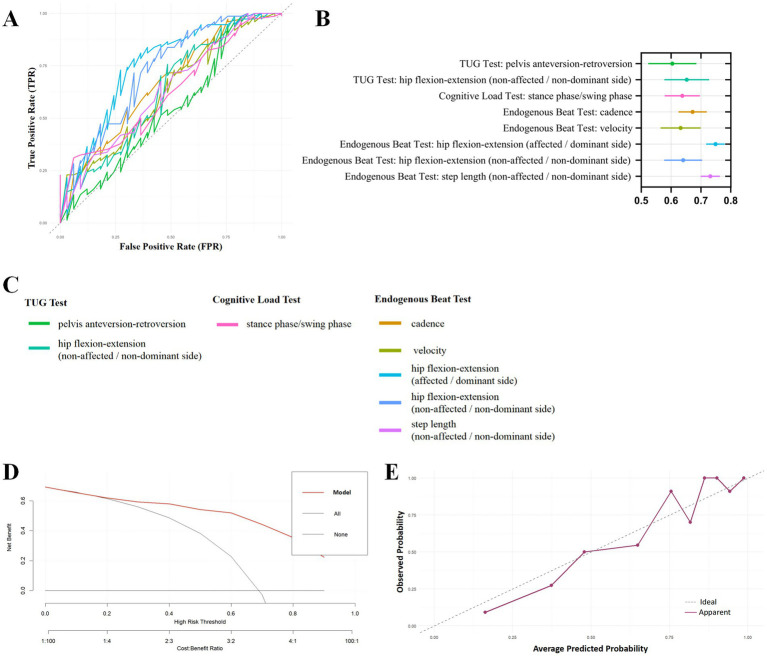
5-fold cross-validated ROC curves, DCA curve, and calibration curve of gait characteristics. ROC: receiver operating characteristic, DCA: decision curve analysis, PD: Parkinson’s disease, MSA-P: the Parkinsonian subtype of multiple system atrophy, TUG: timed up and go. **(A)** 5-fold cross-validated ROC curves of gait characteristics with statistically significant results between PD-FOG and MSA-P patients; **(B)** corresponding AUC and 95% CI in 5-fold cross-validated ROC curves; **(C)** list of corresponding indicators and colors in 5-fold cross-validated ROC curves; **(D)** DCA curve for 5-fold cross-validated ROC curves (set gait characteristic indicators in 5-fold cross-validated ROC curves as a model); **(E)** calibration curve for model of 5-fold cross-validated ROC curves.

The results of 5-fold cross-validated ROC curves (Figures 4A–C) demonstrated that the ROM of pelvis anteversion–retroversion and the ROM of hip flexion–extension on the non-A/non-D side in the TUG test; stance phase/swing phase in the Cognitive Load Test; and cadence, velocity, the ROM of hip flexion–extension of the A/D side, the ROM of hip flexion–extension on the non-A/non-D side, and step length on the non-A/non-D side in the Endogenous Beat Test might possess discriminative capacity for identifying MSA-P patients from PD patients. Among them, the AUC of the ROM of bilateral hip flexion–extension in the Endogenous Beat Test exceeded 0.70, suggesting the most favorable discriminative capacity. Then, we set the gait variables in the 5-fold cross-validated ROC curves as a model to draw the DCA curve (Figure 4D) and the calibration curves (Figure 4E). The DCA curve indicated good clinical utility and a favorable net benefit, and the calibration curves revealed high consistency between actual and predicted probabilities, indicating good predictive performance.

### Sensitivity analysis

To assess whether the bilateral ROM of hip flexion–extension in the Endogenous Beat Test could also distinguish MSA-P patients from PD patients across different subgroups of patients, we conducted several sensitivity analyses. Supplementary Table 10 presents the data features of PD patients and MSA-P patients in the sensitivity analysis, and Table 2 presents the results of the sensitivity analysis. First, we established Models 1–4 based on sex, BMI, UPDRS-III scores, and LEDD, and classified PD patients and MSA-P patients accordingly. Next, we conducted a sensitivity analysis of the bilateral ROM of hip flexion–extension in the Endogenous Beat Test in different models, respectively, to evaluate whether its discriminative capacity to distinguish MSA-P patients from PD patients was consistent across different models. The results of the sensitivity analysis in Models 1–4 all suggested a decent identification ability of the bilateral ROM of hip flexion–extension in the Endogenous Beat Test, which might represent a potential gait-based biomarker to distinguish MSA-P patients from PD patients.

**Table 2:** Sensitivity analysis of bilateral ROM of hip flexion–extension in the endogenous beat test in PD patients and MSA-P patients.

Models	Variables	Unadjusted		IPW-adjusted	
		*P*	OR (95% CI)	*P*	OR (95% CI)
Model 1: Sex	Male	0.06	1.10 (1.00–1.20)	**0.01**	1.15 (0.97–1.36)
	Female	**<0.01**	1.87 (1.50–2.32)	**<0.001**	1.86 (1.30–2.65)
Model 2: BMI	≥25	**<0.01**	1.34 (1.14–1.58)	**<0.001**	1.29 (1.02–1.63)
	<25	0.07	1.21 (1.06–1.38)	**0.01**	1.11 (0.94–1.30)
Model 3: UPDRS-III	≥20	0.06	1.67 (1.41–1.97)	**0.01**	1.59 (1.32–1.92)
	<20	**<0.01**	1.14 (1.00–1.30)	**<0.001**	1.13 (0.93–1.37)
Model 4: LEDD	≥350	0.05	1.17 (1.05–1.32)	**<0.01**	1.18 (1.03–1.36)
	<350	**<0.01**	1.13 (0.86–1.49)	**<0.001**	1.09 (0.79–1.50)

ROM: range of motion, PD: Parkinson’s disease, MSA-P: the parkinsonian subtype of multiple system atrophy, BMI: body mass index, UPDRS-III: Unified Parkinson’s Disease Rating Scale-part III, LEDD: levodopa equivalent daily dose, IPW: inverse probability weighting, N: number, A: average, SD: standard deviation, OR: odds ratio, CI: confidence interval. Model 1: adjusted for age, BMI, symptom duration, UPDRS-III score, LEDD, MMSE, MoCA, HAMD, and HAMA. Model 2: adjusted for sex, age, symptom duration, UPDRS-III score, LEDD, MMSE, MoCA, HAMD, and HAMA. Model 3: adjusted for sex, age, BMI, symptom duration, LEDD, MMSE, MoCA, HAMD, and HAMA. Model 4: adjusted for sex, age, BMI, symptom duration, UPDRS-III score, MMSE, MoCA, HAMD, and HAMA. *p* values with statistical significance are shown in bold.

## Discussion

The main findings of this study could be summarized as follows: (1) According to gait analysis based on wearable sensors, PD patients and MSA-P patients exhibited reduced stride length, reduced velocity, reduced bilateral step length, anterior pelvic tilt, and broadly reduced ROM of the pelvis and the lower limb joints compared to HCs, which might suggest that stiffness was widespread in different motion planes and joints in these two neurodegenerative diseases. (2) When PD patients and MSA-P patients exhibited similar clinical performance and drug dosage, the ROM of hip flexion–extension in the Endogenous Beat Test demonstrated the most favorable discriminative and potential predictive capacity for distinguishing MSA-P patients from PD patients, and the results were stable across different subgroups of patients. (3) The negative impact of the Cognitive Load Test on gait seemed to be far greater than that of the Endogenous Beat Test, and PD patients might be more sensitive to the Endogenous Beat Test than MSA-P patients. (4) Both the Cognitive Load Test and the Endogenous Beat Test exhibited a tendency to reduce the asymmetry of lower limb movement and joint (5) MSA-P patients exhibited a stronger correlation between the gait characteristics and the clinical performance.

PD patients and MSA-P patients exhibited some common gait patterns in our study. The reduced stride length, velocity, and bilateral step length might reflect a significant impairment of the cerebral locomotion network, whose core nodes (including the lateral premotor cortex and the supplementary motor area) and connections with subcortical structures (including the basal ganglia and the cerebellum) have been demonstrated to play a key role in the occurrence of movement disorder diseases such as PD and MSA (Kaoru, 2013). PD patients and MSA-P patients also showed anterior pelvic tilt and reduced ROM of the pelvis, which was broadly consistent with the results of other PD-based studies (Shida et al., 2023). This might be related to factors such as limb stiffness, postural compensation, rhythm-lacking pelvic movement, and increased head-pelvis co-movement, which could significantly increase the risk of falls (Michael et al., 2016). The reduced ROM of lower limb joints in PD patients and MSA-P patients might be closely associated with dysfunction of the basal ganglia motor network caused by dopaminergic depletion and its decreased functional connectivity with the primary motor cortex (M1), thereby contributing to increased muscle rigidity and joint stiffness (Shanshan et al., 2024). Furthermore, degeneration of dopaminergic neurons has been reported to be accompanied by multiple neurotransmitter alterations, including enhanced glutamatergic activity and reduced *γ*-aminobutyric acid (GABA) levels, which may contribute to axial instability and gait disturbance (Divya and Pravir, 2022).

The gait patterns of PD and MSA-P also exhibited many differences. Our study suggested that when PD patients and MSA-P patients have similar clinical performance and drug dosage, PD patients might be more sensitive to the negative effect of the Endogenous Beat Test than MSA-P patients. Previous etiological studies have reported that PD is characterized by selective degeneration of dopaminergic neurons and dopaminergic depletion in the basal ganglia (specifically the substantia nigra compacta), whereas MSA-P is characterized by extensive atrophy and neuronal loss in striatonigral structures and the corticospinal system (Florian et al., 2024). Functional magnetic resonance imaging studies have indicated that regular/musical rhythms may elicit the coupling of the auditory-motor networks, which involves the striatal-cortical loop, including the basal ganglia (specifically the putamen), supplementary motor area, cerebellum, dorsal premotor cortex, and right frontal lobe (Hoddinott and Grahn, 2024). Pathological studies have shown that, as the disease course progresses, MSA-P patients may experience a rapid aggravation of brain structural damage, which may result in a faster decline in responsiveness to rhythmic stimuli compared with PD patients (Florian et al., 2023). In our study, MSA-P patients had approximately 19.4 months shorter symptom duration than PD patients and were still in the early stage of the disease (with an average symptom duration of 16.9 months), which might partially explain why PD patients were more sensitive to the negative effects of the Endogenous Beat Test than MSA-P patients. However, considering the rapidly deteriorating clinical symptoms of MSA-P, when the symptom durations of the two groups of patients were the same or the disease duration was further prolonged, the result might undergo a dramatic change. However, whether MSA-P patients in the advanced stage would still be able to complete the required tests remains an open question.

Our study also suggested that the Cognitive Load Test may have a notable negative impact on gait in PD patients and MSA-P patients. Functional imaging studies have indicated a close association between cognitive function and gait performance, which share common brain areas, including the prefrontal cortex, supplementary motor area, posterior parietal cortex, basal ganglia, and hippocampus (Abhilasha et al., 2023). The coordination of cognitive-motor circuits requires extensive involvement of visuomotor coordination, visuospatial function, attention and executive function, motion imagery and planning, and working memory, which is a complex and finely regulated process (Abhilasha et al., 2023). Some studies also revealed that the Cognitive Load Test could lead to overactivation of the prefrontal cortex, which partially compensates for the malfunctioning of the indirect locomotion pathways and reduced network efficiency (Grazia et al., 2021). There has been a considerable amount of evidence suggesting that cognitive impairment may be one of the most important factors contributing to gait deficits in patients with neurodegenerative diseases, including PD and MSA (Monaghan et al., 2023; Amina et al., 2022). However, the negative impact of cognitive dysfunction in MSA patients on gait may occur much earlier than clinical observations. Some studies have reported that even at the mild cognitive impairment stage, MSA patients have been found to exhibit a more noticeable deterioration of cortical structures (including the frontal-temporo-parietal region), subcortical structures (including the bilateral putamen, left globus pallidus, and left cerebellum), and white matter than PD patients, which may be related to a decline in multiple cognitive domains, especially the attention-executive domain and memory domain (Huize et al., 2024). Furthermore, dysfunction across multiple neurotransmitter networks has been suggested to contribute to cognitive decline in neurodegenerative diseases, among which cholinergic denervation may play the most direct role in the impairment of attention/working memory and executive function (Crowley et al., 2023). In PD patients, acetylcholine levels have been reported to be upregulated in the early stage of the disease course to partially compensate for dopaminergic neuronal loss, while in MSA-P patients, such a compensatory mechanism has not been observed (van der Zee et al., 2022; Gilman et al., 2010).

The three-dimensional gait motion capture system based on wearable sensors used in our study has demonstrated unique advantages in gait analysis. First, compared with traditional gait analysis relying on a controlled laboratory environment (e.g., optical motion capture systems and force plates), this three-dimensional gait motion capture system allows gait evaluation outside the laboratory, which may better reflect real-life conditions (Michela et al., 2025). Second, the gait parameters provided by this system were highly analyzable. Even over short walking distances, this system was still capable of sensitively capturing movement trajectories and dynamically recording spatiotemporal and kinematic parameters, which was user-friendly and convenient for patients with gait disorders. Furthermore, gait parameters from this system were highly consistent with previous studies, confirming the reliability of this system (Lazzaro et al., 2022). Third, this system clearly demonstrated the negative impact of the Cognitive Load Test and the Endogenous Beat Test on gait in PD and MSA-P patients. The negative impact of the Cognitive Load Test on gait performance has been confirmed by numerous gait-related studies, and a consensus has been reached (Monaghan et al., 2023). However, for the Endogenous Beat Test, the results were not uniform in different studies. Some studies found that endogenous cueing could increase gait velocity, cadence, and stride length, as well as reduce walking variability, while others found that the impact of endogenous cueing on gait was far less than that of exogenous cueing (Nascimento et al., 2024). This discrepancy might arise from endogenous rhythm perception dysfunction (mainly related to the striato-pallido-thalamocortical pathway) among patients with parkinsonian syndromes, whereas exogenous rhythmic cueing (mainly related to the cerebello-thalamo-cortical pathway) may compensate for this deficit and facilitate active self-regulation of subjects’ gait rhythm to synchronize with the exogenous rhythm (Elinor et al., 2025b). In conclusion, this gait motion capture system might hold considerable application potential for clinical differential diagnosis and the formulation of individualized rehabilitation strategies in neurodegenerative diseases.

### Limitations

This study has some limitations. First, the sample size was relatively small, which might weaken the statistical power, although our sample size met the requirements for this study. Large-scale multicenter studies and long-term cohort studies are still recommended to verify the results. Second, this study lacked histopathological confirmation, which was a limitation of every study that relied on clinical diagnosis (Charlotte et al., 2018). We included only typical clinical cases to minimize this limitation as much as possible. Third, the sequence of three tasks was fixed (TUG test–Cognitive Load Test–Endogenous Beat Test), and a learning effect could not be excluded. However, this influence seemed to be very limited, as there was no tendency of improvement observed in the Cognitive Load Test and the Endogenous Beat Test compared to the TUG test.

## Conclusion

According to three-dimensional gait analysis based on wearable sensors, when PD patients and MSA-P patients have similar clinical performance and drug dosage, both of them tend to exhibit reduced stride length, reduced velocity, reduced bilateral step length, anterior pelvic tilt, and broadly reduced ROM of the pelvis and the lower limb joints compared to HCs. The negative impact of the Cognitive Load Test on gait seemed to be far greater than that of the Endogenous Beat Test, and PD patients might be more sensitive to the Endogenous Beat Test than MSA-P patients. The ROM of hip flexion–extension in the Endogenous Beat Test demonstrated the most favorable discriminative and potential predictive capacity for distinguishing MSA-P patients from PD patients, and the results were stable across different subgroups of patients. Three-dimensional gait analysis based on wearable sensors might hold considerable application potential for the clinical differential diagnosis of neurodegenerative diseases.

## Data Availability

The raw data supporting the conclusions of this article will be made available by the authors, without undue reservation.
