# Supplementary material for: Potential for distinguishing the parkinsonian subtype of multiple system atrophy from Parkinson’s disease: a three-dimensional gait analysis study
Source: Front Aging Neurosci. 2026 May 7;18:1797960. doi: 10.3389/fnagi.2026.1797960 (PMC13190579; doi:10.3389/fnagi.2026.1797960)
Supplement: Supplementary file 1 [file Data_Sheet_1.DOCX]

**Supplementary-Table 1 Gait Characteristics of PD Patients, MSA-P Patients, and HCs in TUG Test**


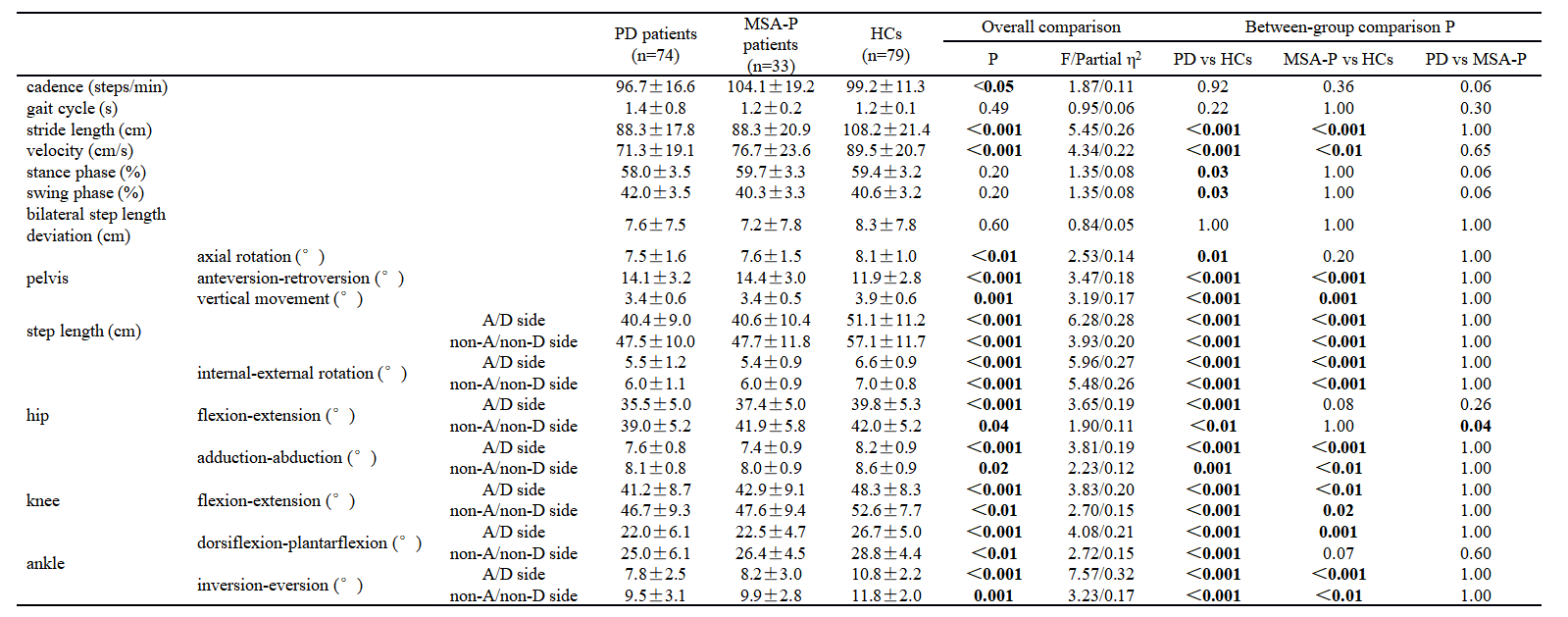


NOTE: PD: Parkinson's disease, MSA-P: the Parkinsonian subtype of multiple system atrophy, HC: health control, TUG: timed up and go, A/D side: affected / dominant side, non-A/non-D side: non-affected / non-dominant side.

MANCOVA (adjusted for group, sex, age, BMI, MMSE, MoCA, HAMD, and HAMA) was used to calculate overall comparisons P, and post-hoc Bonferroni was used to calculate between-group comparison P.

**Supplementary-Table 2 Main Effect Tests for MANCOVA Comparisons in TUG Test**


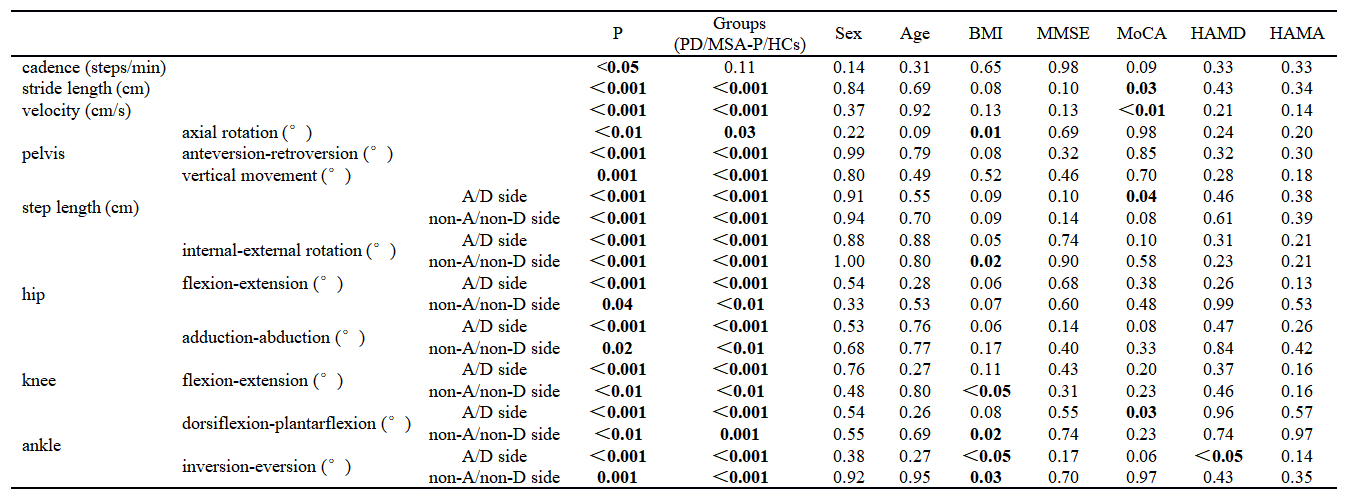


NOTE: PD: Parkinson's disease, MSA-P: the Parkinsonian subtype of multiple system atrophy, HC: health control, TUG: timed up and go, BMI: body mass index, MMSE: Mini-Mental State Examination, MoCA: Montreal Cognitive Assessment, HAMD: Hamilton Depression Scale, HAMA: Hamilton Anxiety Scale, A/D side: affected / dominant side, non-A/non-D side: non-affected / non-dominant side.

This table listed results of main effect tests for MANCOVA comparisons of gait characteristic indicators with statistically differences in TUG test.

**Supplementary-Table 3 Gait Characteristics of PD Patients, MSA-P Patients, and HCs in Cognitive Load Test**


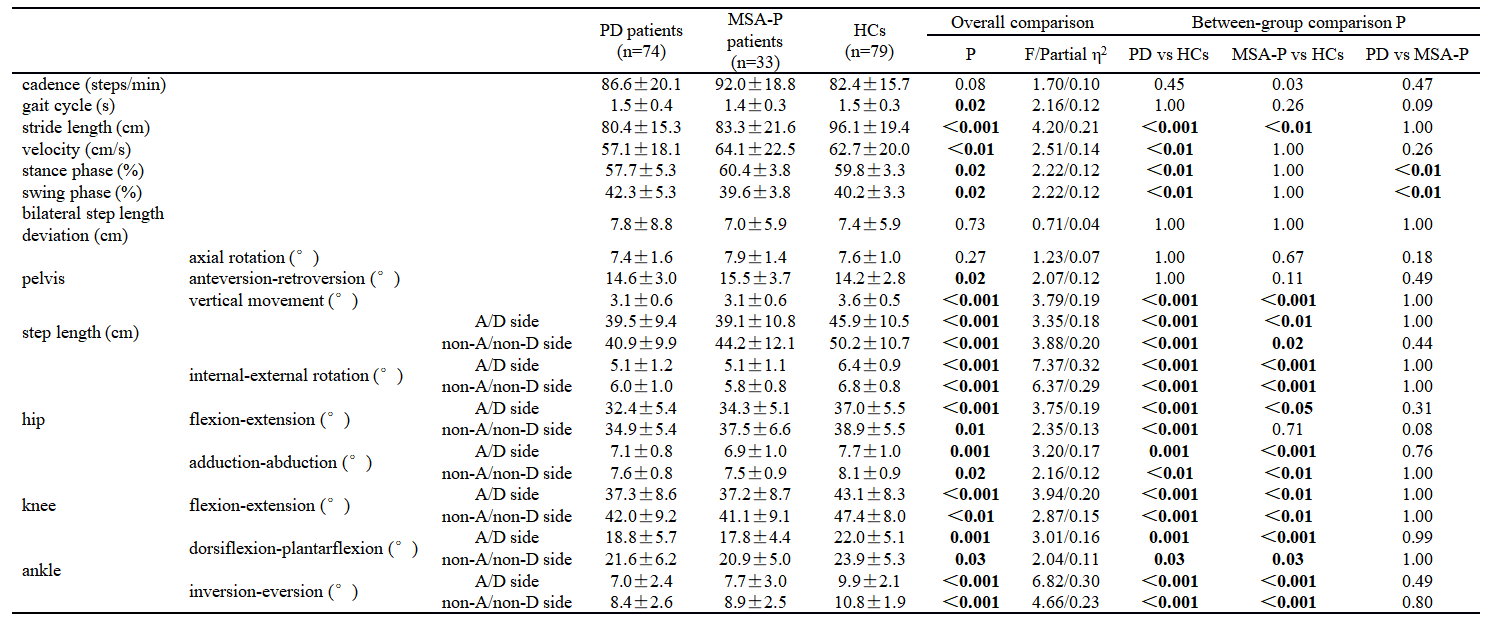


NOTE: PD: Parkinson's disease, MSA-P: the Parkinsonian subtype of multiple system atrophy, HC: health control, A/D side: affected / dominant side, non-A/non-D side: non-affected / non-dominant side.

MANCOVA (adjusted for group, sex, age, BMI, MMSE, MoCA, HAMD, and HAMA) was used to calculate overall comparisons P, and post-hoc Bonferroni was used to calculate between-group comparison P.

**Supplementary-Table 4 Main Effect Tests for MANCOVA Comparisons in Cognitive Load Test**


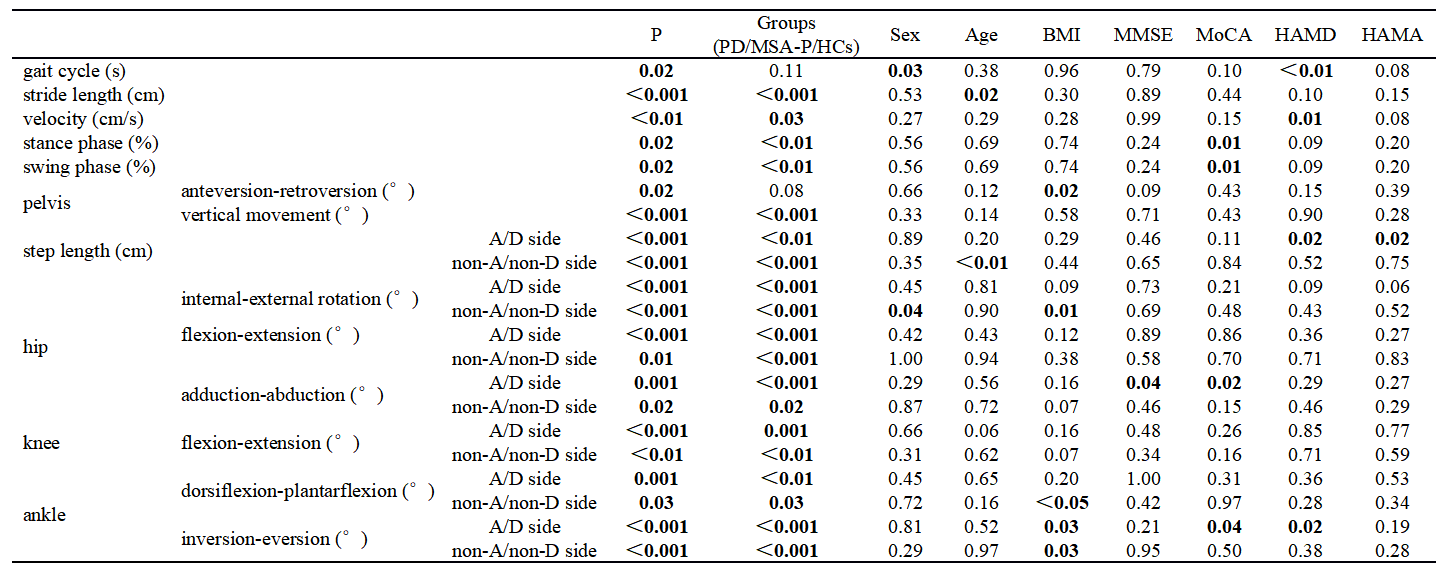


NOTE: PD: Parkinson's disease, MSA-P: the Parkinsonian subtype of multiple system atrophy, HC: health control, BMI: body mass index, MMSE: Mini-Mental State Examination, MoCA: Montreal Cognitive Assessment, HAMD: Hamilton Depression Scale, HAMA: Hamilton Anxiety Scale, A/D side: affected / dominant side, non-A/non-D side: non-affected / non-dominant side.

This table listed results of main effect tests for MANCOVA comparisons of gait characteristic indicators with statistically differences in cognitive load test.

**Supplementary-Table 5 Gait Characteristics of PD Patients, MSA-P Patients, and HCs in Endogenous Beat Test**


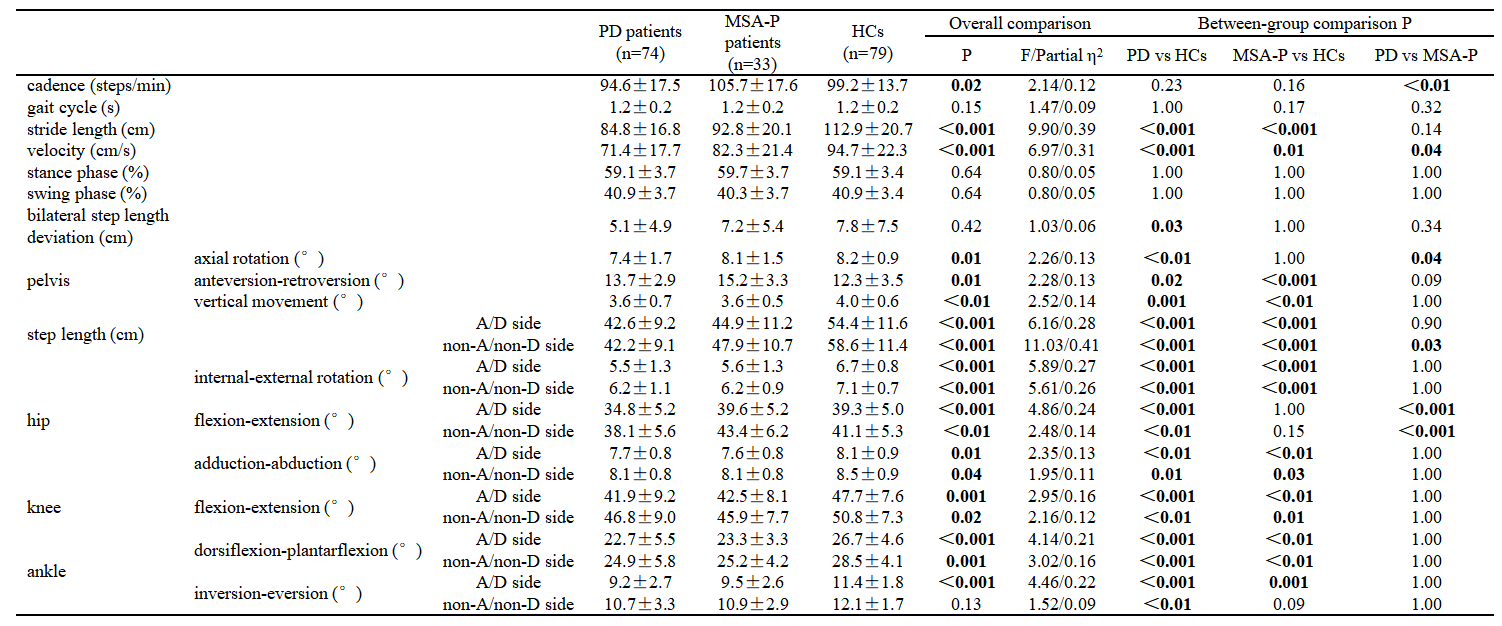


NOTE: PD: Parkinson's disease, MSA-P: the Parkinsonian subtype of multiple system atrophy, HC: health control, A/D side: affected / dominant side, non-A/non-D side: non-affected / non-dominant side.

MANCOVA (adjusted for group, sex, age, BMI, MMSE, MoCA, HAMD, and HAMA) was used to calculate overall comparisons P, and post-hoc Bonferroni was used to calculate between-group comparison P.

**Supplementary-Table 6 Main Effect Tests for MANCOVA Comparisons in Endogenous Beat Test**


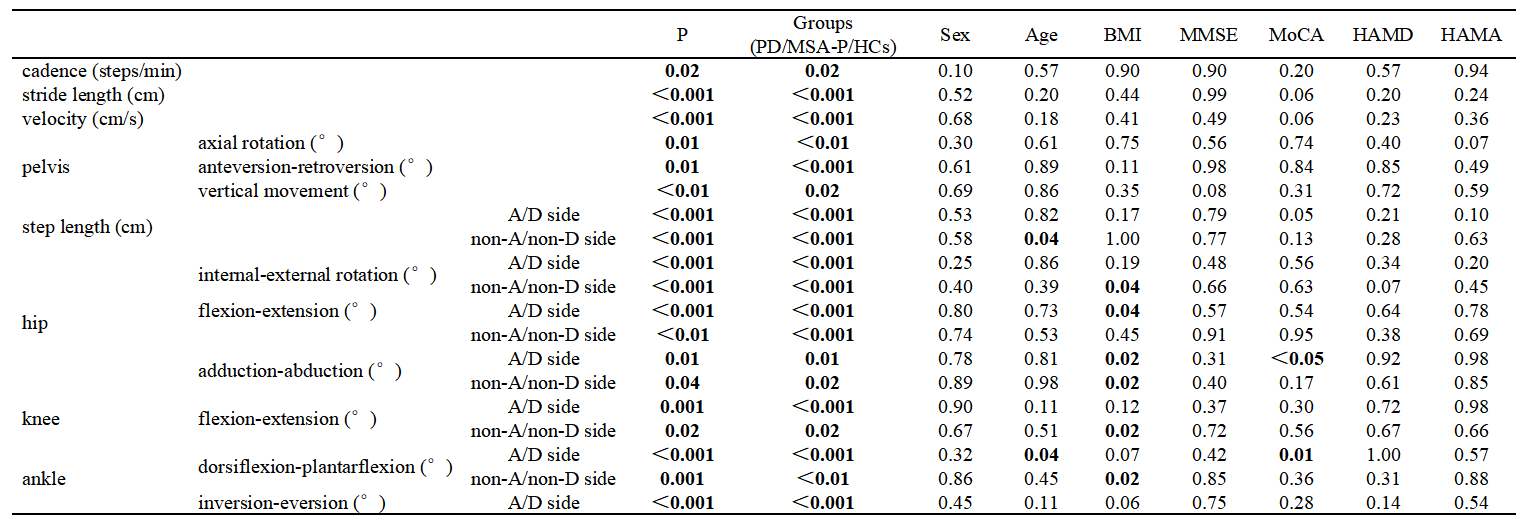


NOTE: PD: Parkinson's disease, MSA-P: the Parkinsonian subtype of multiple system atrophy, HC: health control, BMI: body mass index, MMSE: Mini-Mental State Examination, MoCA: Montreal Cognitive Assessment, HAMD: Hamilton Depression Scale, HAMA: Hamilton Anxiety Scale, A/D side: affected / dominant side, non-A/non-D side: non-affected / non-dominant side.

This table listed results of main effect tests for MANCOVA comparisons of gait characteristic indicators with statistically differences in endogenous beat test.

**Supplementary-Table 7 Comparisons of Gait Characteristics in TUG Test, Cognitive Load Test, and Endogenous Beat Test**


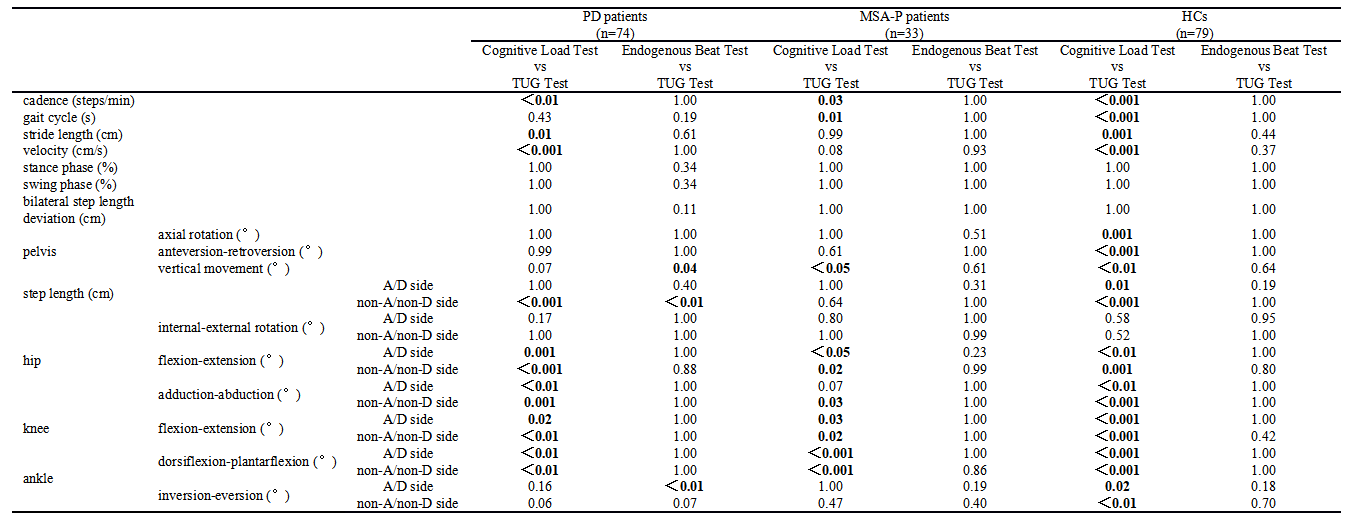


NOTE: PD: Parkinson's disease, MSA-P: the Parkinsonian subtype of multiple system atrophy, HC: health control, TUG: timed up and go, A/D side: affected / dominant side, non-A/non-D side: non-affected / non-dominant side.

ANCOVA was used to calculate overall comparisons P, and post-hoc Bonferroni was used to calculate between-group comparison P.

Set the results of TUG Test as the baseline, this table listed the comparisons between Cognitive Load Test and TUG Test, and Endogenous Beat Test and TUG Test respectively in three groups.

**Supplementary-Table 8 Comparisons of Gait Characteristics between the Affected / Dominant Side and Non-affected /Non-dominant Side in TUG Test, Cognitive Load Test, and Endogenous Beat Test**


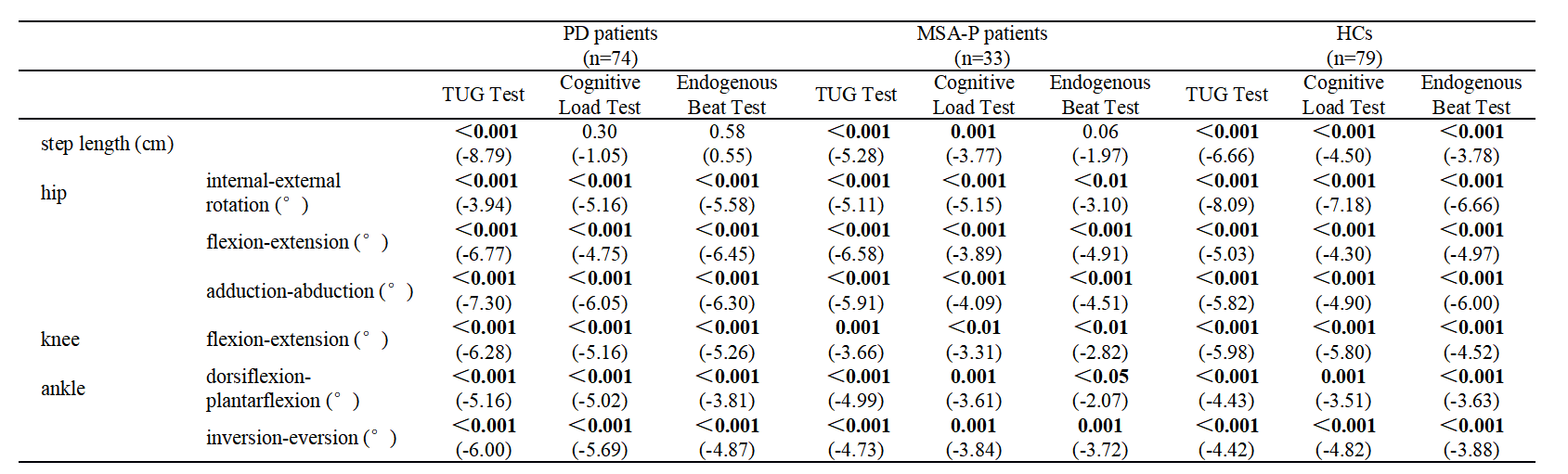


NOTE: PD: Parkinson's disease, MSA-P: the Parkinsonian subtype of multiple system atrophy, HC: health control, TUG: timed up and go.

Independent sample t-tests were used to compared gait variables of the affected / dominant side and non-affected /non-dominant side in three groups.

The data was presented as P(F).

**Supplementary-Table 9 Logistic Univariate and Multivariate Analysis of Gait Characteristics between PD Patients and MSA-P Patients in TUG Test, Cognitive Load Test, and Endogenous Beat Test**


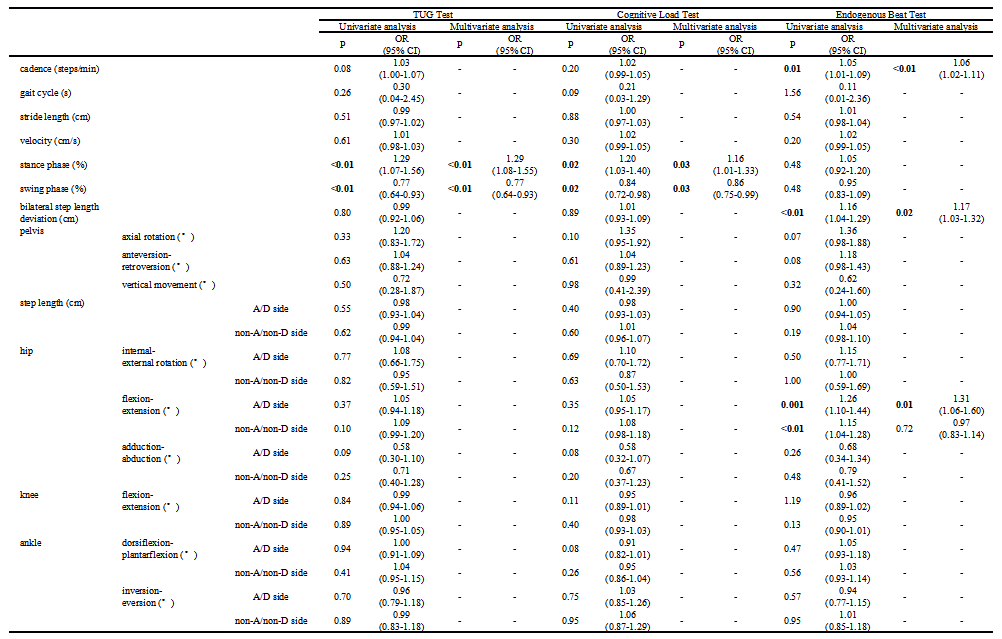


NOTE: PD: Parkinson's disease, MSA-P: the Parkinsonian subtype of multiple system atrophy, OR: odds ratio, CI: confidence interval, TUG: timed up and go, A/D side: affected / dominant side, non-A/non-D side: non-affected / non-dominant side.

This table listed the results of binary logistics regression analysis (adjusted for sex, age, BMI, symptom duration, UPDRS-III score, LEDD, MMSE, MoCA, HAMD, and HAMA) between PD patients and MSA-P patients in TUG Test, Cognitive Load Test, and Endogenous Beat Test.

**Supplementary-Table 10 Demographic and Clinical Features of PD Patients and MSA-P Patients in Sensitivity Analysis**


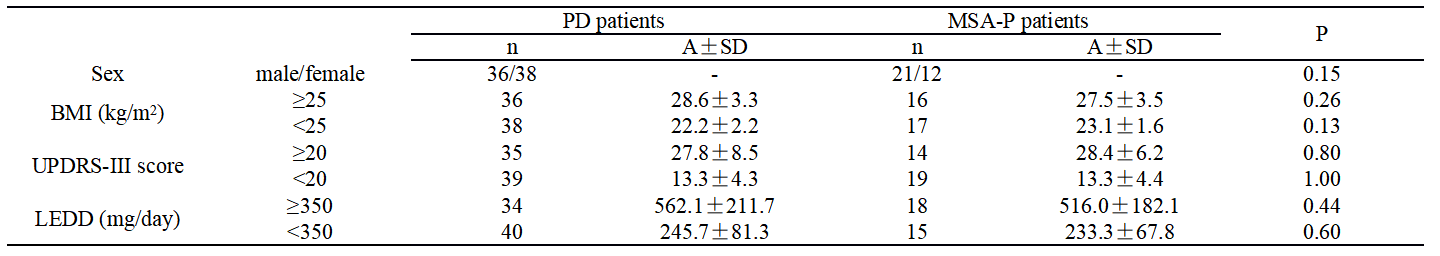


NOTE: PD: Parkinson's disease, MSA-P: the Parkinsonian subtype of multiple system atrophy, A:average, SD:standard deviation, BMI: body mass index, UPDRS-III: Unified Parkinson's Disease Rating Scale-part III, LEDD: levodopa equivalent daily dose.

**Supplementary-Figure 1 Heatmap of Partial Pearson Correlation Coefficient of Gait Characteristics**

**
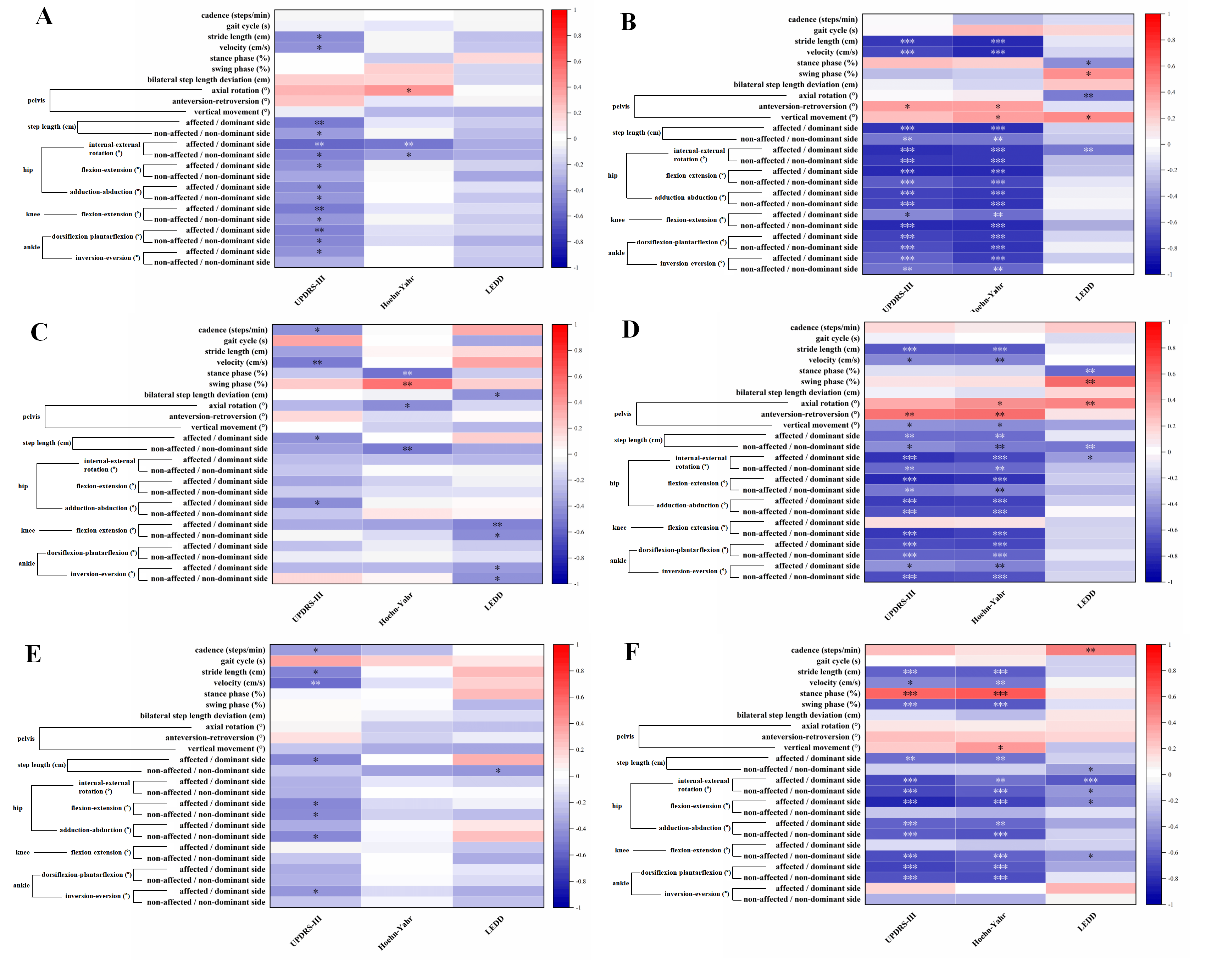
**

NOTE: PD: Parkinson's disease, MSA-P: the Parkinsonian subtype of multiple system atrophy, UPDRS-III: Unified Parkinson's Disease Rating Scale-part III, LEDD: levodopa equivalent daily dose.

Figure 4 showed heatmap of Partial Pearson correlation coefficient (adjusted for sex, age, BMI, symptom duration, MMSE, MoCA, HAMD, and HAMA) of PD patients in TUG Test (A), Cognitive Load Test (C), and Endogenous Beat Test (E), as well as MSA-P patients in TUG Test (B), Cognitive Load Test (D), and Endogenous Beat Test (F) between gait characteristics and clinical features.

*: P<0.05, **: P<0.01, ***: P<0.001.
